# Tumor Microenvironment-triggered Nanosystems as dual-relief Tumor Hypoxia Immunomodulators for enhanced Phototherapy

**DOI:** 10.7150/thno.46076

**Published:** 2020-07-13

**Authors:** Zijun Shen, Junfei Xia, Qingming Ma, Wei Zhu, Zhen Gao, Shangcong Han, Yan Liang, Jie Cao, Yong Sun

**Affiliations:** 1 Department of Pharmaceutics, School of Pharmacy, Qingdao University, Qingdao, 266021, China.; 2 Department of Electrical and Computer Engineering, Tufts University, Medford, MA, 02155, USA.; 3 Department of Pharmacology, School of Pharmacy, Qingdao University, Qingdao, 266021, China.

**Keywords:** Tumor microenvironment, dual-relief hypoxia, enhanced PDT, acute immune response, synergistic anticancer therapy

## Abstract

Photodynamic therapy (PDT) is a promising strategy in cancer treatment that utilizes photosensitizers (PSs) to produce reactive oxygen species (ROS) and eliminate cancer cells under specific wavelength light irradiation. However, special tumor environments, such as those with overexpression of glutathione (GSH), which will consume PDT-mediated ROS, as well as hypoxia in the tumor microenvironment (TME) could lead to ineffective treatment. Moreover, PDT is highly light-dependent and therefore can be hindered in deep tumor cells where light cannot easily penetrate. To solve these problems, we designed oxygen-dual-generating nanosystems MnO_2_@Chitosan-CyI (MCC) for enhanced phototherapy.

**Methods**: The TME-sensitive nanosystems MCC were easily prepared through the self-assembly of iodinated indocyanine green (ICG) derivative CyI and chitosan, after which the MnO_2_ nanoparticles were formed as a shell by electrostatic interaction and Mn-N coordinate bonding.

**Results**: When subjected to NIR irradiation, MCC offered enhanced ROS production and heat generation. Furthermore, once endocytosed, MnO_2_ could not only decrease the level of GSH but also serve as a highly efficient in situ oxygen generator. Meanwhile, heat generation-induced temperature increase accelerated *in vivo* blood flow, which effectively relieved the environmental tumor hypoxia. Furthermore, enhanced PDT triggered an acute immune response, leading to NIR-guided, synergistic PDT/photothermal/immunotherapy capable of eliminating tumors and reducing tumor metastasis.

**Conclusion:** The proposed novel nanosystems represent an important advance in altering TME for improved clinical PDT efficacy, as well as their potential as effective theranostic agents in cancer treatment.

## Introduction

Photodynamic therapy (PDT) is a widely recognized clinical cancer therapeutic strategy owing to its extraordinary merits over conventional chemotherapy, including minimal invasion, excellent spatial specificity, and minimal harm to normal tissues. As an external energy-triggered therapy, PDT typically involves the use of photosensitizing agents, termed photosensitizers (PSs), which expose tissues to a specific light wavelength in order to produce high-level reactive oxygen species (ROS), especially singlet oxygen (^1^O_2_), which in turn eliminate nearby cells 1-4. Moreover, apart from local tumor treatment, PDT has been demonstrated to be capable of inducing immune responses, which may be either immunostimulatory or, in some circumstances, immunosuppressive 5. Particularly, PDT is certified to be effective at stimulating an immune response against locally treated tumors, in which PDT not only can prompt an acute inflammatory response but also can stimulate cells to release secondary inflammatory mediators, thus becoming an encouraging method for distant and metastatic tumor treatment 6-9. However, PDT-induced immune responses are generally mild and not sufficient to eradicate metastatic cells. This is largely attributed to the unique tumor microenvironment (TME). It is widely accepted that the high metabolic rate and high oxygen consumption of the TME, as well as disrupted tumor blood vessels, would lead to hypoxia and an acidification microenvironment. Moreover, oxygen-dependent PDT would result in hypoxia through O_2_ consumption, thus greatly reducing the therapeutic efficiency. At the same time, the overexpressed glutathione (GSH), with a concentration of 1-10 mM in cancer cells, could exhaust ROS generated by PSs, enormously neutralizing the efficacy of PDT 10. In addition, the therapeutic effect of PDT in deep tissue tumors is greatly hindered since most PDTs used in the clinical setting are light-dependent 11. Therefore, it is of central importance to develop intelligent nanosystems that are capable of functioning inside the TME, which not only can improve the oxygen supply and reduce the level of GSH but also can enhance the therapeutic efficacy for deep tumors.

In recent years, various O_2_-evolving agents, such as HbO_2_, catalase, and perfluorocarbon, have been incorporated into PDT nanosystems 12-19. Although these nanosystems have shown potentials for self-supplying oxygen in PDT processes, they still cannot counterbalance the GSH-induced depletion of singlet oxygen. Manganese dioxide (MnO_2_) nanoparticles have been demonstrated to possess high reactivity toward H_2_O_2_ and to produce O_2_ and consume GSH efficiently under TME conditions. They have also been developed as fluorescence quenchers due to their broad absorption spectrum 20. Many groups have designed and constructed MnO_2_ nanoparticle-based nanosystems in order to improve the therapeutic efficacy of PDT 21-36. However, challenges like insufficient and noncontinuous oxygen supply, as well as lack of deep light penetration through living tissues, have not been overcome and therefore limit those developed nanosystems to superficial tumors 27-30, 35, 37. The use of near-infrared light (NIR, 700-1100 nm) and NIR-sensitive PSs can maximize penetration in the tissue and achieve efficient treatment in deep tissue tumors. Small organic molecules are the primary PSs used in the clinical setting due to their convenient synthesis, reproducibility, and biocompatibility. For example, indocyanine green (ICG), which has been approved by the FDA for clinical applications, exhibits photo-to-photodynamic and photo-to-photothermal properties 32, 38-39. However, the generation efficiency of singlet oxygen remains very low.

**Figure 1:**
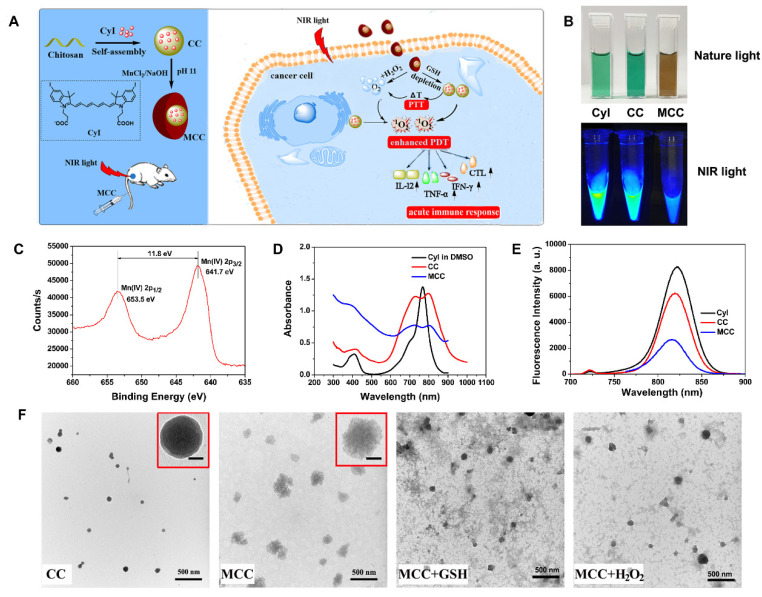
(**A**) Activation mechanism of MCC nanosystems for highly efficient phototherapy and acute immune response. The MCC nanosystems can be prepared by self-assembly of CyI and chitosan, after which the MnO_2_ nanoparticles are formed as a shell by electrostatic interaction and Mn-N coordinate bonding. After intravenous injection, MCC nanosystems can efficiently deliver photosensitizers into tumor cells. Once endocytosed, MCC could be responsive to TME and dual-modulate tumor hypoxia to enhance NIR-guided phototherapy and acute immune response simultaneously in order to combat primary and metastatic tumors; (**B**) Photos of CyI, CC, and MCC in water solution under ambient light and NIR light; (**C**) The XPS spectrum analysis of Mn [IV] 2p peak from MCC; Absorption (**D**) and fluorescence (**E**) spectra of CyI, CC, and MCC, with the concentrations of CyI in DMSO, CC, and MCC are 25 µM, 25 µM, and 15 µM, respectively; (**F**) TEM images of CC and MCC in the presence or absence of 10 mM GSH or 50 µM H_2_O_2_ solution, respectively (bar, 500 nm); Inset photos are enlarged TEM images of CC and MCC (bar in CC, 10 nm; bar in MCC, 50 nm).

The amount of ^1^O_2_ generated by PSs is controlled by the rate of Intersystem Crossing (ISC). It has been reported that the introduction of heavy atoms, such as Br and I, into a molecule can influence the efficiency of ISC, which is known as the heavy-atom effect 40. In our previous work, a nontoxic iodinated derivative of cyanine dye, CyI, with a singlet oxygen quantum yield (Φ_Δ_) of 75%, was successfully synthesized and first used for highly efficient NIR-guided synergistic phototherapy, owing to its enhanced ^1^O_2_ generation and excellent photo-to-photothermal conversion properties 41. Therefore, CyI can be a good candidate NIR PS for intelligent MnO_2_-based nanosystems that are sensitive for TME and deep tissue PDT. Hence, in this study, we designed TME-sensitive nanosystems MnO_2_@Chitosan-CyI (MCC) for highly efficient PDT, which functions by adsorbing MnO_2_ nanoparticles in CyI-modified chitosan nanocomposites (Figure 1A). These nanosystems can be easily prepared through the self-assembly of CyI and chitosan, after which the MnO_2_ nanoparticles are formed as a shell by electrostatic interaction and Mn-N coordinate bonding. Once endocytosed, owing to the advantageous pH/H_2_O_2_/GSH-responsive behavior of MnO_2_, such nanocomposites could not only decrease the level of GSH but also serve as a highly efficient in situ oxygen generator to enhance PDT upon NIR irradiation. Meanwhile, fluorescence recovery of CyI caused by the dissolution of MnO_2_ can provide fluorescence signals, which can be further used for real-time *in vivo* imaging. Most importantly, it was reported that the local hyperthermia created by photothermal therapy (PTT) increases intratumoral blood flow and oxygenation, which is favorable for PDT 42. Therefore, the heat generated by CyI upon appropriate NIR irradiation could not only exhibit photothermal therapy efficacy for tumor sites but also increase oxygen supply to the tumor, further dual-modulating the hypoxia microenvironment to enhance PDT and PDT-induced immune response.

## Results and Discussion

The strategy for the synthesis of MCC nanosystems is shown in Figure 1A. Chitosan possesses great potential in biological applications due to its unique physical and chemical properties, including being rich in terminal amine groups. CyI is a hydrophobic dye that lacks tumor targeting capabilities, which hinders its application in targeted cancer therapy. Using chitosan as a carrier could enhance the loading capacity of CyI to the targeted tissue and extend its retention time in tumors, as well as prevent CyI from self-aggregation in blood circulation. Chitosan-CyI (CC) nanoparticles were first formed via reactions between carboxyl groups of CyI with amino groups of chitosan. MCC nanosystems were then obtained through a biomineralization procedure 19, which induced the formation of metal ions into metal oxide nanoclusters under room temperature. The solution color changed from green to brown during the formation of MCC (Figure 1B).

The chemical state of elemental Mn in the synthesized MCC was evaluated by X-ray photoelectron spectroscopy (XPS) (Figure S1A and S1C). As shown, two characteristic peaks at 653.5 and 641.7 eV were observed, which were corresponding to the Mn (IV) 2p1/2 and Mn (IV) 2p3/2 spin-orbit peaks of MnO_2_, respectively, indicative of the formation of MnO_2_. The obtained MCC had a size distribution of 142 ± 29.1 nm, which is larger than that of CC (57.8 ± 14.6 nm) (Figure S1B). The zeta potential of CC was +34.3 mV, while the potential of MCC was -2.51 mV (Figure S1C). These properties would result in MCC becoming more readily and deeply accumulated in tumors due to the enhanced penetration and retention (EPR) effect, as well as avoiding opsonization and Kupffer cell clearance 43. TEM images in Figure 1F show that the obtained CC and MCC both exhibited spherical structures with uniform sizes of 36.7 nm and 125.9 nm, respectively. Compared with CC, MCC showed a rougher surface, owing to the MnO_2_ coating (inset TEM images in Figure 1F). The size as measured by dynamic light scattering (DLS) was larger than that observed by TEM, which may be due to the shrinkage of nanoparticles during the sample preparation of TEM 44. Next, the UV-Vis-NIR absorption spectrum was measured (Figure 1D). Compared with CC and CyI, MCC showed a much stronger absorbance band centered at 360 nm, which could be associated with the surface plasmon band of colloidal MnO_2_ 35. Fluorescence spectra in Figure 1E showed that the fluorescence intensity of CC was lower than that of CyI in DMSO, which may be attributed to the aggregation of CyI inside water-soluble nanoparticles, causing fluorescence quenching. In addition, the decrease in fluorescence intensity of MCC indicated that MnO_2_ could quench the fluorescence of CyI. Moreover, the stability of MCC was monitored for 30 days, after which size was investigated by DLS (Figure S2). As shown, MCC maintained little change in hydrodynamic diameter during long-term storage, demonstrating MCC's good physical stability.

**Figure 2:**
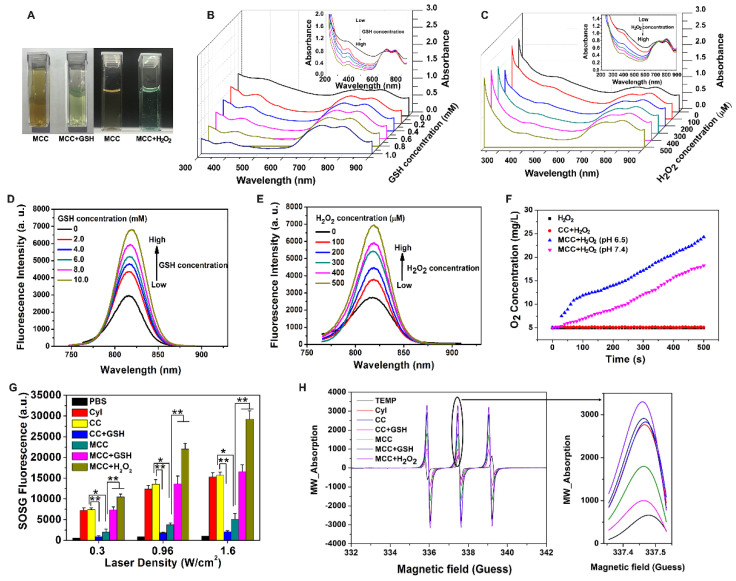
(**A**) Photographs of MCC in the presence of GSH (10 mM) or H_2_O_2_ (50 µM); UV-Vis-NIR absorption spectra of MCC by adding 0-10 mM GSH (**B**) or 0-500 µM H_2_O_2_ (**C**); Fluorescence spectra of MCC by adding 0-10 mM GSH (**D**) or 0-500 µM H_2_O_2_ (**E**); (**F**) Oxygen generation of CC or MCC in 100 µM H_2_O_2_ solutions (pH 6.5 or 7.4); (**G**) ^1^O_2_ generation by PBS, CyI, CC, and MCC with or without 10 mM GSH or 50 µM H_2_O_2_ solution by varying laser power density (808 nm, 0.3, 0.96, or 1.6 W/cm^2^, 3 min); (**H**) ESR analysis of CyI, CC, and MCC with or without 10 mM GSH or 50 µM H_2_O_2_ solution under NIR irradiation (808 nm, 0.96 W/cm^2^).

Considering the acidification of TME and the enhanced H_2_O_2_ concentration in tumor cells, the degradation behavior of MnO_2_ in MCC was systematically studied. After incubating MCC with 50 μM H_2_O_2_ or 10 mM GSH, the degradation of MnO_2_ on the surface of CC resulted in a smoother surface (Figure 1F), which is consistent with the TEM observation results. Meanwhile, the average sizes of MnO_2_ in H_2_O_2_ and GSH, decreased to 40.8 nm and 56.3 nm, respectively, indicating that the deposition of MnO_2_ did not affect the structural integrity of CC. Previous studies have shown that nanoparticles in the range of 30-50 nm exhibit great tumor uptake and excellent penetration capability for poorly permeable tumors to achieve an improved antitumor effect 45. Thus, the designed MnO_2_-coated nanoparticles (~120 nm) with the MnO_2_ shell could be degraded after reacting with H_2_O_2_ and GSH at the tumor site, which makes the particle size (~40 nm) decrease and reach the deep tumor more easily. By tuning the concentration of added H_2_O_2_ or GSH, the color of the solution changed from brown to light green (Figure 2A). Interestingly, obvious oxygen bubbles were observed in the MCC-added H_2_O_2_ solution, demonstrating the excellent capabilities of MnO_2_ to generate oxygen. Then, absorption spectra of MCC after adding different concentrations of GSH or H_2_O_2_ were determined (Figure 2B-C). As shown, the absorbance band of MnO_2_ in MCC gradually decreased when increasing the concentration of H_2_O_2_ or GSH, illustrating that MnO_2_ could be efficiently reduced to Mn^2+^ ions by H_2_O_2_ or GSH, which may be explained by Equation (1) 46:

MnO_2_ + H_2_O_2_ + 2H^+^ = Mn^2+^ + 2H_2_O + O_2_;

MnO_2_ + GSH → Mn^2+^ + GSSG

MnO_2_ nanoparticles have an intense and broad optical absorption spectrum (200-600 nm), making them an efficient broad-spectrum fluorescence quencher for fluorescent dyes such as CyI. The TME-sensitive fluorescence recovery (Figure 2D-E) showed that increasing the concentration of GSH or H_2_O_2_ would result in a corresponding increase in fluorescence intensity, and there was no obvious change in the maximum emission wavelength. These results further demonstrated that MCC could react with GSH or H_2_O_2_ to provide a turn-on fluorescence signal for monitoring the delivery of the nanosystems. As is well-known, under neutral and acidic conditions, MnO_2_ either catalyzes the decomposition of H_2_O_2_ to produce O_2_ or reacts with H_2_O_2_ and H^+^ to generate O_2_. Thus, the oxygen generated by MCC in H_2_O_2_ solutions was evaluated using an oxygen probe (JPBJ-608 portable dissolved oxygen meters, Shanghai REX Instrument Factory) under different pH values (6.5 and 7.4). As shown in Figure 2F, significant amounts of O_2_ were sustainably produced by MCC in H_2_O_2_ (100 μM) solution under both pH values, with more oxygen generated at pH 6.5. In contrast, negligible oxygen was produced in blank H_2_O_2_ or H_2_O_2_ blended with CC solutions, further demonstrating that MnO_2_ could potentially catalyze oxygenation in the presence of H_2_O_2_ to attenuate tumor hypoxia and thereby modulate the tumor microenvironment.

^1^O_2_ generation from MCC was then evaluated by SOSG, which is exclusively selective toward ^1^O_2_, but not to other ROS or compounds 47. The singlet oxygen quantum yield (Φ_Δ_) of CyI was calculated to be 75%, higher than that of commercially used PS Ce6 (Φ_Δ_=65%) 41. After conjugation with chitosan, the SOSG fluorescence intensity was comparable with that of CyI, and the SOSG fluorescence intensity was dramatically decreased after adding 10 mM GSH to CC solution, further confirming that CyI could be consumed by GSH (Figure 2G). Furthermore, compared with free CyI, the MCC nanosystems presented obvious lower SOSG fluorescence intensity upon NIR irradiation, suggesting that MnO_2_ nano-coating could efficiently prevent CyI from playing roles outside the cells and thus reducing the side effects of PDT. In contrast, after mimicking the TME by adjusting the solution's pH to 6.5, adding 100 μM H_2_O_2_, or solely adding 10 mM GSH, would result in increased fluorescence intensity of SOSG up to 5.5 times, due to the degradation of MnO_2_ by the reaction with H_2_O_2_ or GSH. Among samples with MCC and mimicking TME, SOSG fluorescence intensity in H_2_O_2_ solutions was higher than that in GSH solutions, which may be due to the oxygen generation in H_2_O_2_/H^+^ solutions, as these can result in enhanced singlet oxygen generation. To confirm the singlet oxygen generation of MCC under TME conditions, we further used ESR analysis for ^1^O_2_ generation. As depicted in Figure 2H, the ESR signals for CyI, CC, and MCC, with or without 10 mM GSH or 50 μM H_2_O_2_ solution, clearly displayed a 1:1:1 triplet signal characteristic. This is consistent with those for 2,2,6,6-tetramethylpiperidine-N-oxyl (TEMPO). Particularly, after adding H_2_O_2_ or GSH, signal intensity was found to be significantly higher than that of MCC, which is consistent with the results detected by SOSG, further demonstrating that the PDT efficiency could be greatly enhanced by MnO_2_-nanocoating CyI-chitosan systems in the TME consisting of abundant H_2_O_2_ and GSH.

It is known that CyI is the iodinated derivative of Cy7 41. Heavy atom effect refers to a well-known photophysical phenomenon that the introduction of heavy atoms like bromine and iodine usually makes the spin-orbit coupling of heavy atoms stronger, thus promoting intra-system channeling, quenching fluorescence, and increasing the phosphorescence yield. Therefore, after introducing heavy atomic iodine to Cy7, the singlet oxygen generation of CyI is significantly enhanced while the fluorescence intensity is reduced, with a fluorescence quantum yield of 48% (Figure S3A). For the photothermal conversion efficiency, the results of CyI and Cy7 were both calculated by Equation S1 (see Supporting Information) and the results were comparable; moreover, as demonstrated in Figure S3B, the temperature changes of CyI and Cy7 during 8 min are also comparable, indicating that the additional of iodine into Cy7 show more influence on the fluorescence intensity of CyI than on its photothermal conversion efficiency. As shown in Figure S3A, the fluorescence intensity was reduced after iodine modification, with a fluorescence quantum yield of 48%. However, the photothermal effect was comparable with Cy7 (Figure S3B)*.* According to our previously reported method 41, the photothermal conversion efficiency of MCC under 808 nm laser irradiation was measured to be 50.21%, comparable to that of CyI (49.15%), indicating that MCC possessed PTT behavior upon NIR irradiation*.* The photothermal stability of MCC was investigated by recording the temperature changes of MCC (50 µg/mL CyI-equiv) exposed to the laser under different irradiation times (Figure S3C). It is well known that when the temperature is above 43 °C, efficient photothermal therapy can be exhibited 48. As shown, when the power density of the laser beam was lowered to 0.3 W/cm^2^, no photothermal effect was detected; however, under the power of 0.96 W/cm^2^ and 1.6 W/cm^2^, the temperature of MCC first increased with the irradiation time and gradually decreased after reaching a peak. It should be mentioned that the temperature could be maintained above 43 °C after 7 min NIR irradiation, which is sufficient for PTT treatment. The photothermal properties of MCC nanosystems were also evaluated by adjusting laser power density in TME-mimic solution (Figure S3D). As expected, the temperature profile of MCC had no obvious changes compared with that of water solution, implying that MCC possessed dual PDT/PTT behavior in the TME. This may be explained by the fact that MnO_2_ coating does not affect the vibronic relaxation of CyI in the excited energy state.

**Figure 3:**
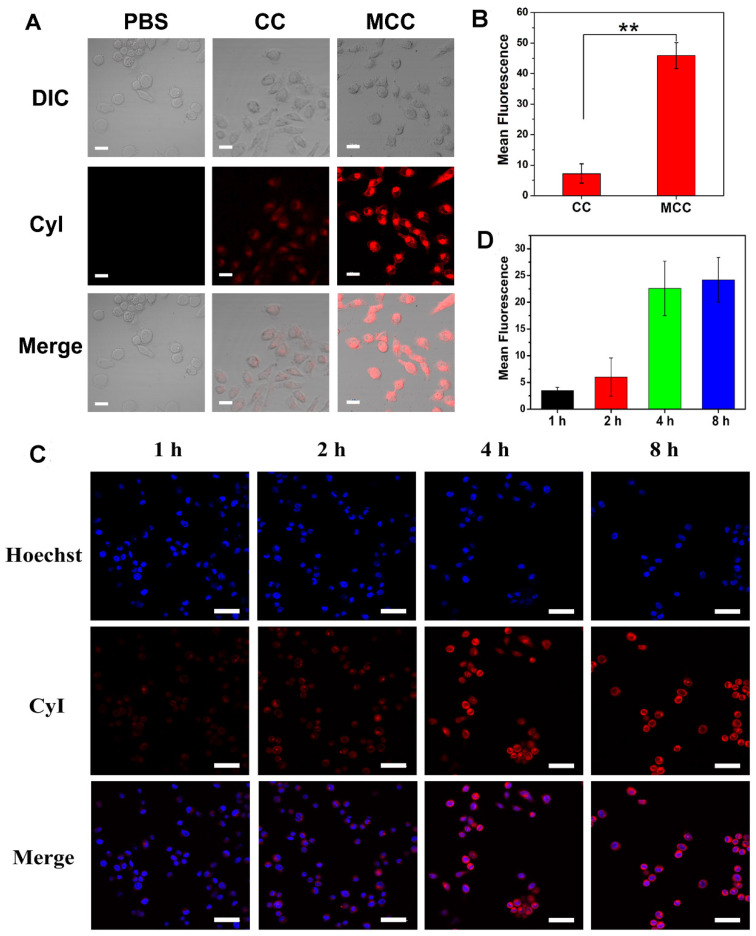
(**A**) Confocal fluorescence images of 4T1 cells incubated with CC and MCC with the same CyI concentration; bar, 20 µm; (**B**) Semi-quantitative analysis of the fluorescence intensity of CC and MCC; (**C**) Confocal fluorescence images of 4T1 cells incubated with MCC for 1, 2, 4, and 8 h, with the nuclei stained with Hoechst; bar, 50 µm; (**D**) Semi-quantitative analysis of the fluorescence intensity of MCC incubated in 4T1 cells for 1, 2, 4, and 8 h.

As dark cytotoxicity is one of the key factors affecting the safety and efficiency of PDT, MTT assay was first performed in both a human normal liver cell line (L-02 cells) and murine mammary carcinoma cell line (4T1 cells) to investigate the cytotoxicity of MCC nanosystems (Figure S4). As expected, both CC and MCC showed no obvious cytotoxicity even at the highest CyI concentration of 200 μM after 48 h incubation, indicating that neither the introduction of iodine atoms nor inorganic materials of MnO_2_ render detectable toxicity. The cellular uptake efficacy of MCC was then determined in 4T1 cells, with CC as the control (Figure 3A-B). CLSM images showed that, compared with CC-treated cells, MCC-treated cells exhibited much higher fluorescence intensity, illustrating that the formation of MnO_2_ nanoparticles could increase the internalization of CyI. Before* in vitro* therapeutic study, the time point at which NIR laser irradiation was performed was optimized by observing the fluorescence images of MCC incubated with 4T1 cells for 1, 2, 4, and 8 h (Figure 3C). The semi-quantitative analysis of fluorescence intensity is shown in Figure 3D. Blue images showed cell nucleus stained with Hoechst in order to locate cells, and red images showed CyI fluorescence in MCC. The fluorescence intensity of CyI gradually increased within 4 h and leveled off for the next 4 h. Hence, we chose to apply NIR laser for cell irradiation after 4 h incubation for phototherapy. Images also showed that MCC was mainly distributed in the cytoplasm, giving evidence that MnO_2_ was mainly reduced by GSH in the cytoplasm. These results demonstrated that MCC could be efficiently taken up by 4T1 cells and used for further phototherapy and fluorescence imaging.

**Figure 4:**
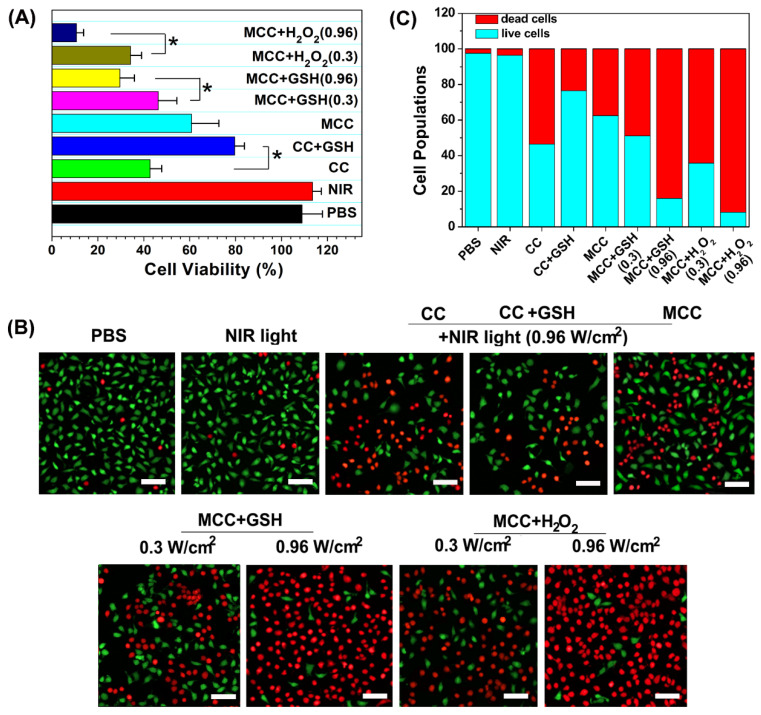
(**A**) Cell viabilities of CC or MCC regardless of the presence of GSH or H_2_O_2_ under NIR irradiation (0.3 or 0.96 W cm^-2^, 3 min); (**B**) Confocal images of 4T1 cells treated with different samples costained with Calcein AM/PI, in which green fluorescence images (ex/em: 490/515 nm) and red fluorescence images (ex/em: 535/615 nm) represent live and dead cells, respectively. Bar, 20 µm; (**C**) Semi-quantitative count of cell populations from AM/PI images.

To identify the phototherapy efficacy of the MCC nanosystems, an MTT assay and viability assay using calcine acetoxymethyl ester/propidium iodide (calcein-AM/PI) were conducted for the developed nanosystems with 808 nm light irradiation. Since MCC could generate both ^1^O_2_ and heat inside cells upon NIR irradiation, we evaluated their synergistic PDT and PTT effect by monitoring their 4T1 cell-killing ability as a function of MCC concentration and laser intensity using MTT assays. The CI index was calculated in accordance with previous methods 5. When the laser power density is higher than 0.3 W/cm^2^, MCC exerts a PTT effect accompanied by the occurrence of a PDT effect. The photothermal conversion efficiency was comparable to another NIR dye, cypate, while the PDT effect of cypate was quite low and could be ignored. Therefore, we chose cypate instead of CyI to construct MCCypate to detect the IC50 when it was used for PTT treatment alone. As shown in Figure S5, the IC50s for the individual use of PDT and PTT were 290 μg/ml and 214 μg/ml, respectively. When using the combined treatment, IC50 reduced to 84.77 μg/ml, and the CI value was calculated to be 0.68. It is known that when the value of CI is below 1, the combined effect of the two therapies indicates a synergistic effect, confirming that the combined photothermal therapy and photodynamic therapy had a synergistic effect, rather than simple superposition. Furthermore, it is well known that the endogenous GSH and H_2_O_2_ can destroy MnO_2_, owing to the overexpressed GSH and H_2_O_2_ in cancer cells 49. To further determine whether GSH or H_2_O_2_ plays a more vital role in inducing the degradation of MnO_2_, we externally added GSH or H_2_O_2_ in the medium. After incubation, some of the GSH or H_2_O_2_ would enter the 4T1 cells through the cell membrane to mimic the endogenous TME. As shown in Figure 4A, CC and MCC both showed anticancer efficacy under NIR irradiation (42.5 ± 5.6% cell death for CC, green bar; 60.8 ± 11.9% cell death for MCC, cyan bar). Interestingly, we noticed that adding GSH (10 mM) could dramatically enhance the cell viability of CC (79.6 ± 4.8%, blue bar) but decrease the viability of cells treated with MCC (46.3 ± 8.1%, magenta bar, and 29.6 ± 6.3%, yellow bar). The treatment by MCC with additional H_2_O_2_ added offered the most effective killing of cancer cells, especially under higher NIR irradiation (89.3 ± 3.1% cell death, dark blue bar). Meanwhile, in both MCC groups with additional GSH and H_2_O_2_, MCC showed better anticancer efficacy with higher laser irradiation. In contrast, only NIR laser irradiation exhibited no significant cell death (red bar), implying that the NIR laser itself was harmless. AM/PI staining results in Figure 4B and C were similar to the above results, in which green fluorescence images and red fluorescence images represented live and dead cells, respectively.

Then, to understand the mechanism of *in vitro* phototherapy of MCC in TME-mimic solution, the PTT and PDT effects of MCC were investigated. The temperature profiles and thermal images, which were recorded instantly using a real-time IR thermal imaging system of the cells under NIR irradiation, are shown in Figures S6 and 5E. With a starting temperature of 26.8 ºC, MCC-pretreated cells (either in GSH or H_2_O_2_-supplemented solution with 808 nm and 0.96 W/cm^2^) could reach up to 53.1 ºC and 55.9 ºC, respectively, much higher than temperatures of non-transfected 4T1 cells (32.3 ºC) and solely NIR-treated cell pellets (34.7 ºC). In contrast, MCC-pretreated cells in TME-mimic solutions with lower laser power density (0.3 W/cm^2^) only showed 4 ºC temperature elevation after 3 min irradiation, reaching 38.7 ºC for GSH-supplemented solution and 36.9 ºC for H_2_O_2_-supplemented solution, implying that MCC's photo-to-photothermal effect could be controlled by differing irradiation laser power.

**Figure 5:**
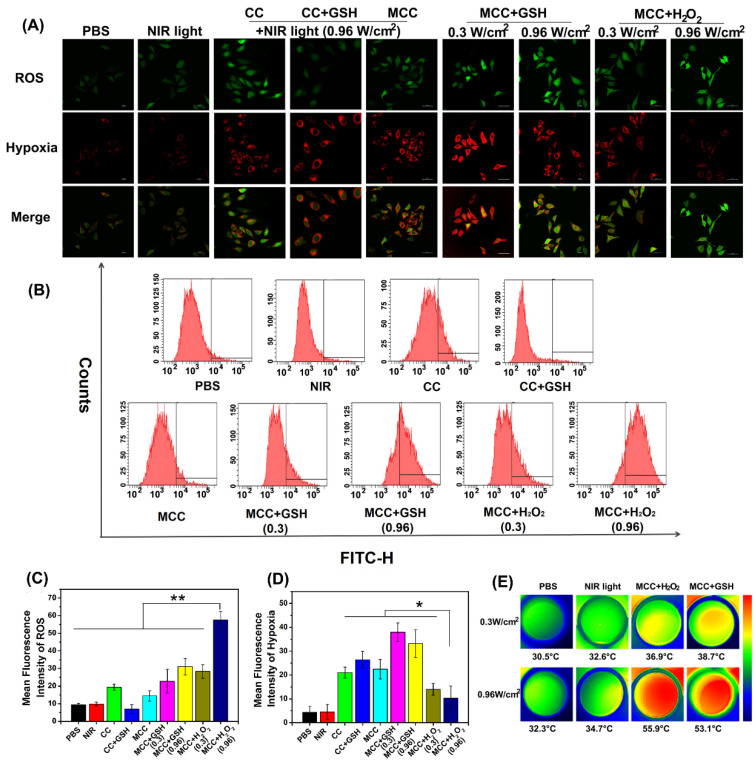
(**A**) Confocal images of 4T1 cells treated with CC or MCC in the presence or absence of GSH or H_2_O_2_ costained with hypoxia/oxidative stress detection probes under NIR irradiation (808 nm, 0.3 or 0.96 W/cm^2^). Green fluorescence represents intracellular ROS generation, while red fluorescence represents hypoxia production; (**B**) The flow cytometric analysis of ROS generation in 4T1 cells cultured with different samples under NIR light irradiation; (**C, D**) Semi-quantitative of fluorescence intensity from A (n =4). Data are shown as mean ± SD; *p < 0.05; **p < 0.01; (**E**) Thermal images of 4T1 cells under laser irradiation (808 nm, 0.3 or 0.96 W cm^-2^, 3 min) after different treatments.

MCC-mediated, light-triggered intracellular ROS generation and hypoxia levels were detected by hypoxia/oxidative stress ROS/hypoxia probes using the confocal laser scanning microscope (Figure 5A). The flow quantitative results and semi-quantities of fluorescence intensity are shown in Figure 5B-D. The hypoxia probe could convert a nitro group to hydroxylamine (NHOH) and amino (NH_2_) via the nitroreductase presented in hypoxic cells, and showed red fluorescence (596/670 nm). The oxidative stress detection probe could react directly with a wide range of reactive species and showed green fluorescence (490/525 nm). As shown, compared with CC, MCC without GSH or H_2_O_2_ showed lower ROS generation, consistent with our PDT evaluation in solutions, which likely resulted because the MnO_2_ nano-coating could efficiently inhibit SOG from interacting with CyI. However, after adding GSH, the ROS generation of CC dramatically decreased, which is evidence of GSH acting as a ROS revenger in 4T1 cells. Meanwhile, adding GSH to MCC-incubated cells enhanced ROS generation, suggesting that MnO_2_ could deplete GSH. Moreover, just as with MCC-GSH, adding H_2_O_2_ to MCC-incubated cells enhanced ROS generation, illustrating that MnO_2_ was reduced to Mn^2+^ ions, and CyI was released for efficient PDT. By increasing the irradiation laser power density, the ROS generation of MCC in TME-mimic solution was correspondingly enhanced. In comparison with control cells and cells treated only with NIR light, cells treated with CC or MCC with or without GSH solution showed a significantly increased level of hypoxia (red), indicating that CyI-mediated PDT consumed O_2_. In contrast, the red fluorescence intensity in the MCC-H_2_O_2_-treated group was apparently lower, implying that the relief from hypoxia by MCC was due to oxygen generation. Specifically, the fluorescence intensity of MCC-H_2_O_2_ or MCC-GSH under 0.96 W/cm^2^ laser power density was much weaker than that under 0.3 W/cm^2^, which may be owing to the fact that under higher laser power, MCC-GSH exhibited a PTT effect and could generate local hyperthermia to accelerate the blood flow and relieve tumor hypoxia to a certain extent (Figure 5E). Therefore, MCC seems to be a useful TME-triggered phototherapy nanosystem, from which we draw the conclusion that the remarkable *in vitro* antitumor efficacy of MCC in TME mimic solution was due to the following advantages: (i) MCC could effectively prevent ROS consumption by reacting with GSH in 4T1 cells for enhanced PDT; (ii) MCC could react with H_2_O_2_ in the 4T1 cells to generate O_2_ to relief hypoxia for efficient PDT; (iii) under higher laser irradiation power, elevated temperature generated by MCC could accelerate oxygen generation and exhibit the enhanced PTT/PDT synergistic effect.

**Figure 6:**
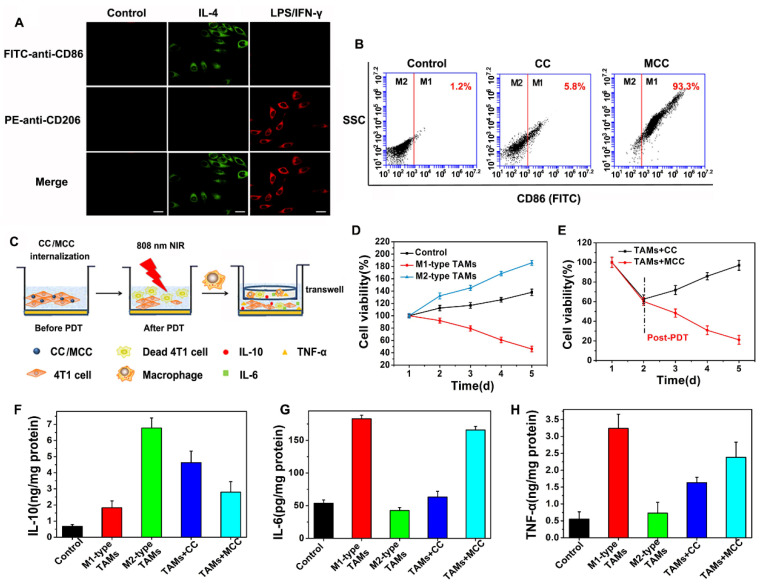
(**A**) Confocal microscope images of macrophages with M0 (monocyte), M1, and M2 phenotype upon IL-4 and LPS/IFN-γ stimulation, and immunostaining with FITC-anti-CD86 or PE-anti-CD206. Green: FITC (Ex: 488 nm, Em: 510 nm); red: PE (Ex: 543 nm, Em: 580 nm). Scale bars: 50 µm; (**B**) FCM analysis of the reprogramed M1-type macrophages from M2-type (TAMs) after incubation with CC and MCC; (**C**) Scheme of inhibition of 4T1 tumor cells after incubation with TAMs upon CC or MCC stimulation; (**D**) Cell viability of 4T1 tumor cells co-cultured with M1-type TAMs or M2-type TAMs; (**E**) Cell viability of 4T1 tumor cells during post-PDT period co-cultured with TAMs incubated with CC or MCC. Quantitative cytokine analysis of pro-tumor IL-10 (**F**), anti-tumor IL-6 (**G**), and TNF-α (**H**) in different groups.

There have been a large number of studies showing that tumor-associated macrophages (TAMs) play a key role in PDT-mediated tumor immunotherapy, where macrophages are described as two distinct populations: the classic activated proinflammatory (M1) phenotype (attacking intruders) and the alternatively activated anti-inflammatory (M2) phenotype (healing damage). Recent research found that TAMs can be regulated as M2 phenotypic cells in hypoxic conditions 50. Considering the ability of our nanoparticles to improve tumor hypoxia, we investigated the immunological role of MCC *in vitro*. M1-like and alternate M2-like activated macrophage models were successfully established using lipopolysaccharide (LPS)/interferon-γ (IFN-γ) and interleukin 4 (IL-4) to stimulate macrophage RAW264.7 lines (M0) in the initial mouse monocytes, respectively. M1 and M2 were labeled with CD86 and CD206, respectively, and confocal imaging analysis was performed with FITC and PE-labeled antibodies (Figure 6A).

Upon successful establishment of M2 type TAMs, we added CC or MCC to the culture in a type M2 environment in order to investigate whether the oxygen-supplemented MCC could reverse type M1 macrophages with flow cytometry (FCM) after staining with FITC-anti-CD86. As shown in Figure 6B, few fluorescence intensity changes were detected in the control and CC treatment groups, indicating that CC had little effect on the macrophage phenotype. However, after being cultured with MCC, the fluorescence signal of FITC-anti-CD86 increased significantly (93.3%), which may be due to increased oxygen supply after adding MnO_2_, reducing the number of M2 macrophages and converting them to M1 type TAMs. These results suggested that MCC could be used as a suitable immunomodulator for antitumor therapy.

Next, we investigated the survival ability of macrophages with different phenotypes in tumor cells and the quantity of relevant immune factors under different conditions through transwell experiments. The experimental scheme is shown in Figure 6C. We co-cultured 4T1 tumor cells with M1 or M2-type TAMs to detect their antagonism/promoting tumor cell potency, as shown in Figure 6D. Compared with the control group, when cells were incubated with M2 type TAMs, their cell survival increased significantly (about 1.5-fold increase), confirming the tumor-promoting effect of M2-type TAMs. When cultured with M1 type, cell viability decreased significantly, indicating the antitumor effect of M1 type TAMs. After confirming the effect of macrophages with different phenotypes on tumor cell growth, we investigated the cell survival effect of TAMs after CC or MCC treatment. As shown in Figure 6E, the viability of cells treated with TAMs+MCC was significantly decreased (21.1%) on the fifth day after PDT irradiation, while the tumor cells after TAMs+CC treatment still showed recurrence, indicating that the MCC-increased oxygen content decreased transition to M2-type macrophages, thus effectively inhibiting the proliferation of residual tumor cells after treatment.

Moreover, we investigated the number of cytokines (IL-6, IL-10, and TNF-α) secreted by macrophages in different activation states. M2 TAMs are known to produce pro-tumor and cytoprotective cytokines (e.g., IL-10) to promote tissue repair, while M1 types secrete anti-tumor cytokines (e.g., IL-6 and TNF-α) to inhibit tumor growth. As shown in Figure 6F, compared with the control group, the IL-10 content was significantly increased when incubated in the TAMs of type M2, but decreased when treated with MCC. However, the cytokines of IL-6 and TNF-α were highly expressed after treatment with TAMs+MCC, and their expression was only slightly lower than the M1-type TAM equivalent (Figure 6G and H), further demonstrating that MCC can inhibit tumor cell proliferation and recurrence by recombining the TAM phenotype, resulting in anti-tumor cytokine secretion and antitumor immunotherapy.

**Figure 7:**
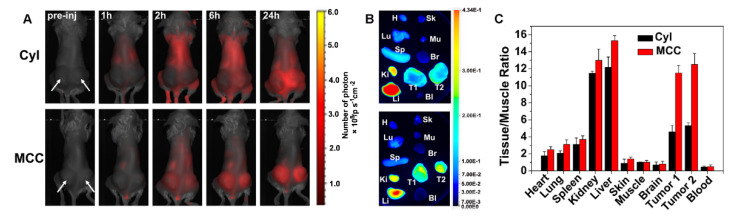
(**A**) *In vivo* fluorescence images of 4T1 tumor-bearing mice intravenously injected with Cyl or MCC at different times. Arrows indicate the tumor's location; (**B**) *Ex vivo* fluorescence images of tumors and main organs isolated from those mice at 24 h post-injection; H, Sk, Lu, Mu, Sp, Br, Ki, Li, Bl, T1, and T2 stand for heart, skin, lung, muscle, spleen, brain, kidney, liver, blood, tumor 1, and tumor 2, respectively; (**C**) Semi-quantitative analysis of *ex vivo* fluorescence images for different organs (24 h post-injection).

*In vitro* therapeutic results of MCC in 4T1 cells motivated us to investigate the *in vivo* phototherapy efficacy. It was anticipated that the prepared MCC nanosystems with a size of ~142 nm could preferentially localize at tumor sites due to an enhanced EPR effect. To prove this hypothesis, we administered MCC (1.5 mg/kg equivalent to CyI) to 4T1 tumor-bearing mice via tail-vein injection and measured its real-time biodistribution and tumor accumulation at various time points (Figure 7A). Mice bearing 4T1 tumors injected with only CyI were used as controls. As shown, a visual identification of tumor sites was available after 2 h injection and retained up to 24 h. In contrast, weak fluorescence of CyI was observed in tumor sites in control mice at 6 h post-injection. It has been reported that some fluorescence dyes are able to accumulate in tumors, which is consistent with our previous findings 44. The mice were sacrificed at 24 h post-injection, and the tumors as well as main organs were isolated for imaging, as shown in Figure 7B. The semi-quantitative analysis of the tissue/muscle (T/M) ratio is plotted in Figure 7C. As shown, a higher level of tumor accumulation for MCC was observed than for CyI, which demonstrated that MCC could preferentially localize at tumor sites. Furthermore, the liver and kidney showed higher fluorescence intensity than other normal organs, indicating that the nanosystems were primarily metabolized by the liver and excreted by the kidneys.

Subsequently, the pharmacokinetics of the plasma and tissues were studied. The mice were intravenously injected with MCC, and ~30 µL of blood was collected at different time points (1 h, 2 h, 4 h, 6h, 8 h, 12 h, and 24 h). Meanwhile, the heart, liver, spleen, lungs, and kidneys were selected according to the predetermined time points and analyzed by UV. The curve of average plasma concentration in Figure S7 showed that MCC was not observed in plasma after 8 h post-injection, demonstrating that MCC could be quickly removed from the plasma. The analysis of tissue distribution in Figure S7 indicated that MCC gathered in the liver and kidneys after intravenous injection and reached a peak at 8 h and 6 h, respectively. MCC could then be cleared from the mice after 24 h, further confirming that MCC was mainly metabolized by the liver and kidneys. Above all, the high degree of tumor accumulation and distribution of MCC showed great promises for imaging-guided, effective phototherapy of tumors.

**Figure 8:**
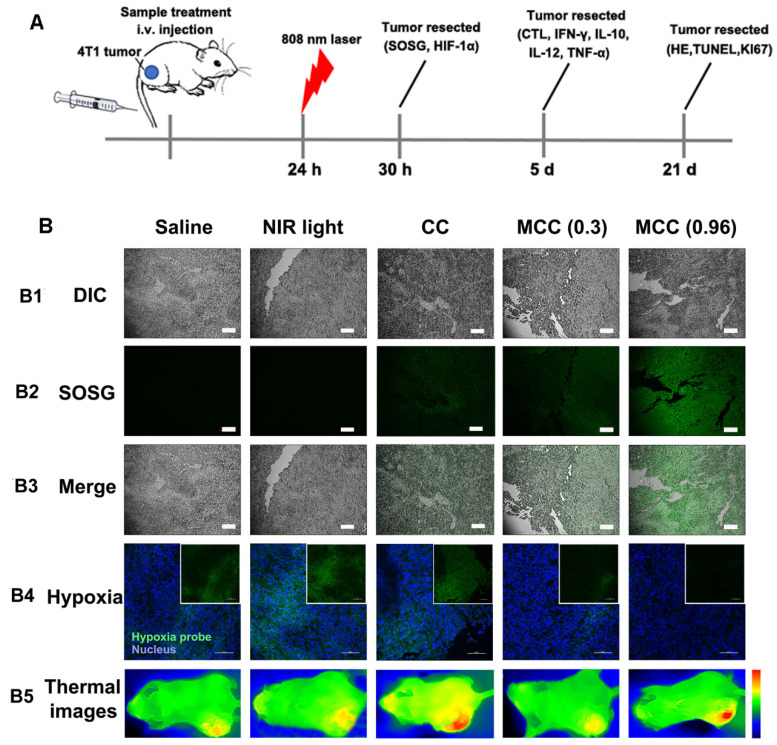
(**A**) The therapeutic processes of MCC in Balb/c mice bearing 4T1 tumors. All mice were intravenously injected once for therapy. NIR laser was applied at 24 h post-injection (808 nm, 0.3 or 0.96 W/cm2, 3 min; (**B**) B1-B3: Confocal images of tumor sections stained with SOSG after intramural injection of CC or MCC plus NIR irradiation. Scale bars represent 100 µm; B4: Representative immunofluorescence images of tumor slices after hypoxia staining. Green and red fluorescence represent hypoxic areas and nuclei, respectively; Scale bars represent 100 µm; B5: Near-infrared thermography of mice treated with different samples under laser irradiation.

For *in vivo* evaluation of MCC-modulated tumor hypoxia for enhanced phototherapy, Balb/c mice bearing 4T1 tumors were randomly divided into five groups (n = 5 per group): (1) saline without laser irradiation, as control group; (2) NIR laser only, also as control group; (3) CC (with CyI 1.5 mg/kg) with laser irradiation, as PTT-modulated tumor hypoxia for enhanced PDT group; (4) MCC with lower laser irradiation (0.3 W/cm^2^), as MnO_2_-modulated tumor hypoxia for enhanced PDT group; (5) MCC with higher laser irradiation (0.96 W/cm^2^), as dual modality-modulated tumor hypoxia for enhanced PDT group. The therapeutic processes are shown in Figure 8A. Singlet oxygen generated by MCC plus NIR laser was first determined by the fluorescence of SOSG in tumor sections (Figure 8B1-3). As shown, tumors treated with CC or MCC plus NIR laser showed obvious green fluorescence compared with the control groups, implying that ^1^O_2_ production can be facilitated by CyI. Semi-quantitative fluorescence intensities of SOSG in tumors are shown in Figure S8. As expected, tumors treated with MCC plus NIR laser exhibited much higher fluorescence intensities than CC, demonstrating that the MnO_2_ nano-coating in PSs could be used for increased PDT efficacy. Moreover, MCC-treated groups with higher laser power density led to more ^1^O_2_, implying that MCC modulation of hypoxia as well as depletion of GSH could enhance the *in vivo* PDT effect. The temperature profiles and thermal images of tumors are shown in Figures S9 and 8B5. In CC and MCC groups plus higher laser irradiation, the tumor temperature could reach up to 46-48 °C, which was sufficient for irreversible tumor ablation. Consistent with *in vitro* evaluation, temperature in tumors treated with MCC plus lower power density (0.3 W/cm^2^) only showed 2 °C elevation, which had no photothermal effect.

In order to explore the ability of MCC to relieve tumor hypoxia, an immunofluorescence assay was conducted using a hypoxyprobe (pimonidazole hydrochloride) on tumor slices extracted from mice 30 h post-injection. The anoxic zone and the nucleus were stained with anti-pimonidazole antibody (green) and DAPI (blue), respectively (Figure 8B4). As shown, compared with control groups, the CC-treated group showed significant green fluorescence (pimonidazole stained hypoxia) in the tumor region upon NIR irradiation, which proves that hypoxia was aggravated. In contrast, the MCC-treated group exhibited significantly reduced green fluorescence, demonstrating that tumor hypoxia was relieved, which may be explained by the high reactivity of MnO_2_ toward endogenous H_2_O_2_ to produce O_2_. Moreover, the MCC-treated group with higher laser power density showed much weaker green fluorescence, further indicating that the elevated temperature induced by MCC could accelerate blood flow, resulting in increased oxygen generation for highly efficient PDT. Such MnO_2_-coating nanosystems could efficiently enhance oxygenation in the TME, which would have a potential for reducing the hypoxia-associated PDT resistance.

**Figure 9:**
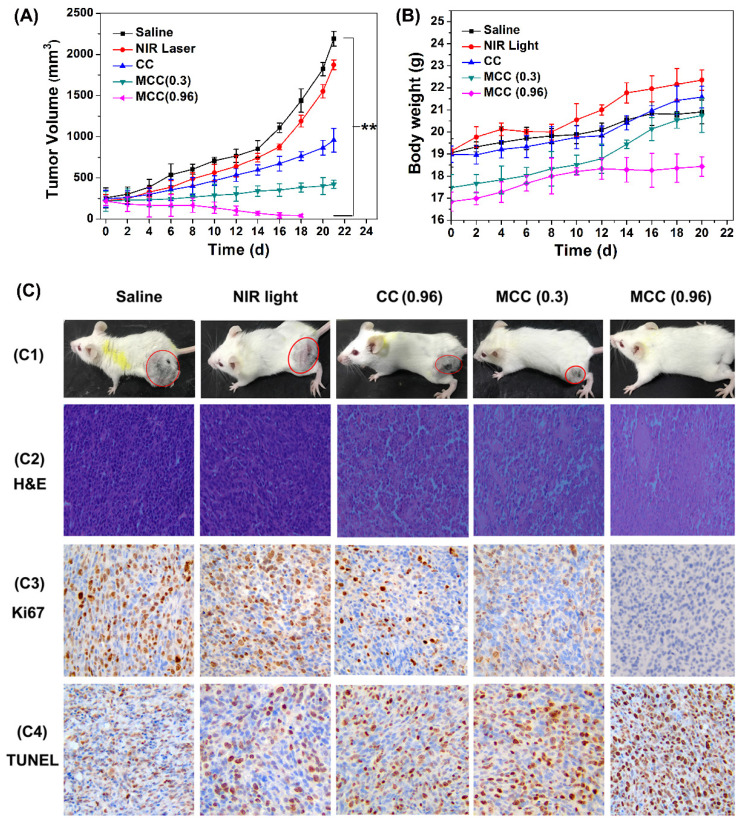
The tumor growth curves (**A**) and body weight changes (**B**) of mice after various treatments; (**C**) C1: The photos of mice after treatment for 21 d; C2-C4: The images of tumors stained with H&E, Ki67, and TUNEL from each group (image magnification is 200×).

The tumor volumes and body weight of mice were measured every other day (Figure 9A-B). As expected, there was no discernible difference in tumor volume between the NIR laser group and the control saline group, while a significant reduction in tumor volume was observed in all other treatments. Compared with the CC group, mice treated with MCC exhibited a stronger inhibitory effect and a dose-dependent manner in which higher laser irradiation almost achieved complete tumor regression. Furthermore, long-term monitoring revealed that there was no recurrence in MCC groups treated with 0.96 W/cm^2^ laser in the following 20 days. In addition, the body weights of all CyI-based groups of mice displayed no discernible decreases during the treatment period, suggesting a high level of biosafety for these treatments. After 21 days of treatment, a representative mouse was taken from each group and photographed for its tumor (Figure 9C1). Meanwhile, remaining mice were dissected and pictured for the excised tumors (Figure S10). MCC-treated group showed excellent anticancer efficacy. Next, histological analysis was performed by H&E staining, Ki67 staining, and TUNEL staining (Figure 9C2-4). No tissue damage or obvious cell proliferation was found in the tumors of control groups. However, significant nuclear lysis and tumor necrosis were visible in the tumors treated by MCC plus NIR laser, especially with higher laser dosage. On the contrary, tumors treated with CC under NIR irradiation showed less nuclear lysis and fewer apoptotic cells, further confirming the enhanced synergistic PDT/PTT efficacy mediated by MCC.

**Figure 10:**
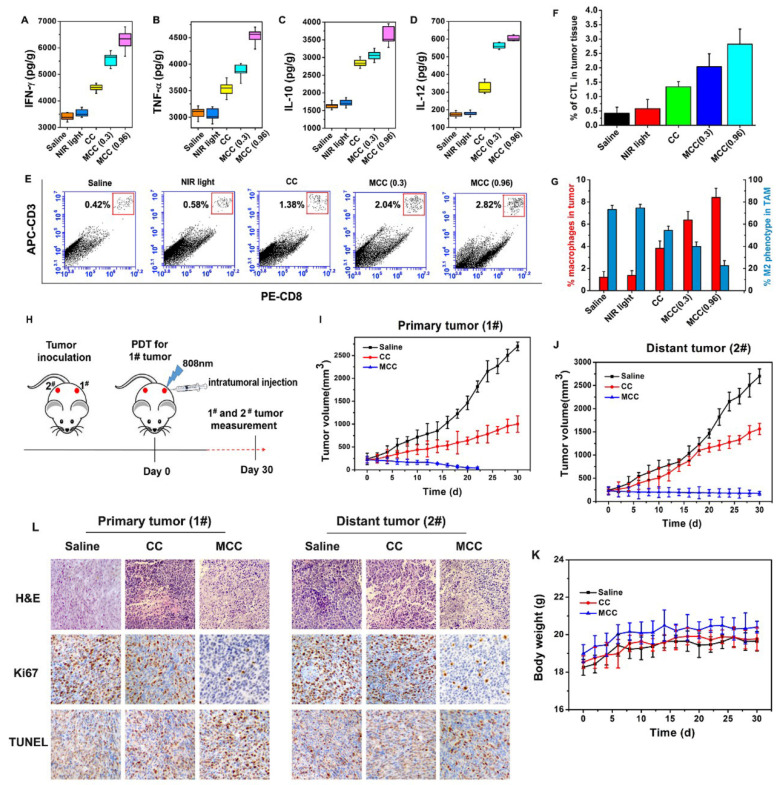
The immune factor concentrations of IFN-γ (**A**), TNF-α (**B**), IL-10 (**C**), and IL-12 (**D**) in tumors; (**E**) Flow cytometry data of cytotoxic T lymphocyte (CTL) infiltration in tumors. CD3^+^ and CD8^+^ cells were defined as CTLs; (**F**) CTL quantification of flow cytometry results; (**G**) Macrophage infiltration and polarization within tumors after various treatments. (**H**) The therapeutic processes of MCC in bilateral subcutaneous 4T1 tumor model. Tumor 1# was designated “primary tumor” and treated with CC or MCC upon NIR irradiation, while Tumor 2# was designated “metastasis tumor” without treatment; (**I**) Growth curves for the primary tumors; (**J**) Growth curves for the distal tumors; (**K**) Body weight changes of mice under the different treatments. (**L**) The images of H&E, Ki67 and TUNEL for dual-tumor tissue slices of saline, CC and MCC groups (image magnification is 200×).

The ideal treatment for tumors is not only to cause local tumor eradication but also to activate the systemic immune response for anticancer therapy, by which the body can effectively eliminate distant metastasis of cancer cells and exert a minimal level of toxicity to normal tissues. After treatment with MCC and laser irradiation, acute immune response is supposed to be activated. To verify the hypothesis, the levels of immune factors, including tumor necrosis factor-α (TNF-α), interferon-γ (IFN-γ), interleukin-12 (IL-12), and interleukin-10 (IL-10) in tumor tissues treated by MCC were detected by ELISA kits. As shown in Figure 10A-D, the levels of TNF-α, IFN-γ, and IL-12 in the groups treated with CC and MCC were significantly increased compared with the levels in the control group, indicating that PDT-induced innate immunity had been activated, accompanied by an acute inflammatory response. Among these groups, the production of immune factors in the CC-treated group was lower due to insufficient synergistic therapy, which was not conducive to the eradication of the tumor. Meanwhile, in the MCC-treated group, the secretion of IL-10 was higher than that in other groups. However, even though IL-10, as a cytokine that causes inflammation inhibition and immunosuppression, can inhibit the synthesis of IL-12, reduce the amount of TNF-α and IFN-γ, and hinder the occurrence of an immune response, this spontaneous immune counterion did not completely prevent anti-tumor efficacy 51, 52.

Furthermore, cytotoxic T lymphocyte (CTL) is a special type of T cell that secretes various cytokines to participate in immune response. CTL levels were measured by flow cytometry in different treatment groups (Figure 10E and F), and cells were stained with anti-CD3-APC and anti-CD8-PE for CTL infiltration. It can be seen that the infiltration rate of CTL in the MCC group was more significantly upregulated after 0.96W/cm^2^ laser irradiation than the CC group, further indicating that MnO_2_-modified MCC could enhance PDT therapy, induce the release of tumor-associated proinflammatory mediators, and activate CD4+ helper T and CD8+ cytotoxic cells to produce immune responses.

Additionally, a large number of studies have shown that TAM plays a significant role in tumor immunotherapy. We investigated the TAM changes in tumors, which were quantified by flow cytometry after five days of treatment (Figure 10G). Interestingly, compared with the saline group, macrophage infiltration was significantly enhanced in mice tumors after caudal vein injection of MCC (0.96 W/cm^2^) from 1.21% to 8.41%. This occurred in conjunction with a largely reduced population of M2 phenotype TAMs, from 73.2% to 22.5% among total TAMs. Studies showed that MnO_2_ itself can induce a certain degree of TAM polarization 53, and the developed MCC samples demonstrated better results in inducing polarization. These results suggest that cell death by MCC phototherapy can trigger inflammatory responses and inflammatory cytokine production, thus triggering the recruitment of cytotoxic T lymphocytes, which could further induce tumor cell death.

To further investigate whether the immunological responses triggered by PDT after MCC treatment would be able to inhibit the growth of tumor cells at metastatic sites, we conducted experiments to treat a bilateral tumor model. First, 10^6^ 4T1 tumor cells were subcutaneously inoculated into both the left and right flanks of each BABL/c mouse. The tumors in the left flanks, as primary tumors (1#), were designed for MCC-based PDT therapy, and the distant tumors in the right flanks (2#) were designed without direct treatment as an artificial model of abscopal tumors (Figure 10H). When both tumors reached ∼100 mm^3^, we divided these mice into three groups: (1) saline, (2) CC with NIR irradiation, and (3) MCC with NIR irradiation. For each tumor on the left flank of a mouse in groups 2 and 3, 50 μL of CC or MCC (with CyI 1.5 mg/kg) was intratumorally injected and exposed to the 808 nm NIR laser at 0.96 W/cm^2^ for 3 min. The tumor sizes on both sides and body weight were closely monitored afterward. In the cell study, MCC was found to reduce the number of M2 macrophages and convert them to M1 type TAMs without NIR irradiation, which may be attributable to the increased oxygen supply after addition MnO_2_. As shown in Figure 7, MCC can accumulate in both tumors via tail-vein injection. To avoid the immune effect of MCC on the distant tumor, we choose to intratumorally inject the samples into the primary tumor rather than perform tail-vein injection. As shown in Figure 10I, for primary tumors, CC and MCC under irradiation could reduce the tumor growth, and MCC showed a stronger inhibitory effect that is consistent with our previous results. As mentioned above (Figure 10A-G), while treatment by CC under NIR irradiation could trigger a mild immune response, it could only partially delay the tumor growth at the distant site (Figures 10J and S11). In contrast, after treatment with MCC upon NIR irradiation, the size of the distant tumor gradually shrank, indicating that treatment with MCC could not only completely eliminate primary tumors but also strongly inhibit the growth of distant tumors. In addition, the body weights of CC- or MCC-treated groups of mice displayed no discernible decreases during the treatment period, further demonstrating the biosafety of these treatments (Figure 10K).

Histological analysis of both primary and distant tumors was performed using H&E staining, Ki67 staining, and TUNEL staining. As shown in Figure 10L, no tissue damage or obvious cell proliferation was found in either type of tumors of control group. In contrast, for primary tumors, obvious nuclear lysis and tumor necrosis were detectable in the tumors treated with MCC, while the tumors treated with CC showed less tissue damage and fewer apoptotic cells. Distant tumors in MCC and CC groups showed a certain degree of tissue damage and necrosis, and tumors in the MCC group exhibited much more severe structural damage and more apoptotic cells than the CC group, which may be due to the systemic immune response triggered by PDT. These results together suggest that our designed iodinated cyanine dye-based nanosystems would offer a strong synergistic antitumor immunological effect to inhibit the growth of tumor cells, even for cells without direct treatment.

## Conclusion

In conclusion, we have successfully developed TME-sensitive functional nanosystems based on iodinated cyanine dyes capable of providing dual-relief hypoxia for NIR-guided, enhanced synergistic PDT/PTT/immunotherapy. Both *in vitro* and *in vivo* therapeutic results demonstrated that the developed nanosystems exhibit excellent anticancer efficacy, which we concluded for four major reasons: 1) MCC improved the hydrophility of PSs and efficiently delivered them to tumor sites by EPR effect, enhancing their accumulation in tumors. 2) After being endocytosed, MnO_2_ coated on the exterior of the nanosystems was sensitive to pH/H_2_O_2_/GSH TME, resulting in decreased GSH level and serving as a highly efficient in situ oxygen generator to enhance PDT upon NIR irradiation. 3) After the degradation of MnO_2_, the released CyI exhibited both enhanced photo-to-photodynamic conversion as well as photo-to-photothermal conversion. The resultant elevated temperature accelerated blood flow to further relieve the environmental hypoxia and kill cancerous cells to achieve a synergistic PDT/PTT effect. 4) Enhanced PDT triggered acute immune responses, further inhibiting tumor cells and reducing tumor metastasis. This novel biocompatible and biodegradable TME-responsive MCC nanosystem overcame some of the key challenges in conventional PDT tumor treatment and demonstrated great promise as a smart, multifunctional nanotherapeutics platform capable of dual TME amelioration for better treatment outcomes.

## Materials and Methods

### Materials

NIR dye CyI (MW 776.5) was prepared in our laboratory. Chitosan was purchased from Aokang Biotechnology Co. Ltd. (Shandong, China), with deacetylation degrees of 90% and average molecular weight (MW) of 10 kDa. Anhydrous manganese chloride (MnCl_2_) was purchased from Yuanye Biotechnology Co. Ltd. (Shanghai, China). L-glutathione (GSH) and H_2_O_2_ were purchased from Sigma-Aldrich (St. Louis, MO, USA). Singlet Oxygen Sensor Green (SOSG), 1,3-diphenylisobenzofuran (DPBF), methylene blue (MB), Methyl thiazolyltetrazolium (MTT), nuclear staining dye (Hoechst 33342), Calcein AM/PI assay kit, Annexin V-FITC/PI apoptosis staining kit were purchased from Solarbio (Beijing, China). Hypoxyprobe™-1 plus kits was purchased from Hypoxyprobe Inc. (Burlington, USA). Hypoxia/oxidative stress detection kit was purchased from Enzo LifeSciences (New York, USA). ELISA test kit of IFN-γ, IL-10, IL-12, TNF-α and CTL were purchased from Biolegend (San Diego, USA). Immune factors were detected by enzyme label analyzer (RaytoRT-6100, Rayto Co. Ltd, China). All other analytical reagent grade chemical reagents used in the study were commercially acquired from Chemical Reagent Company (Qingdao, China).

Mouse breast cancer cells (4T1) and human liver normal cells (L-02) were purchased from American Type Culture Collection (ATCC, Manassas, VA, USA). Cells were cultured in Dulbecco's modified eagle medium (DMEM, Hyclone, USA) with 10% fetal bovine serum (Solarbio, Beijing, China) and 1% penicillin/streptomycin (Solarbio, Beijing, China) at 37 °C in a 5% CO_2_ atmosphere. BALB/c mice (half male and half female) used in this study were purchased from Daren Laboratory Animal Co. Ltd (Qingdao, China), which were 6-8 weeks old and weighed about 16-21 g. All animal experiments were carried out in compliance with the Animal Management Rules of the Ministry of Health of the People's Republic of China (document no. 55, 2001) and were approved by the Animal Care Ethics Committee of Qingdao University (Qingdao, China).

### Preparation of Chitosan-CyI (CC)

Simply, CyI (5 mg) was firstly dissolved in 2 mL anhydrous DMF and reacted with DCC/NHS catalyst systems (molar ratio of CyI: DCC: NHS = 1: 1.5: 1.5) under continuous stirring in the dark for 4 h at room temperature. Then, chitosan (molar ratio of CyI: chitosan = 10:1) was dissolved in 1 mL 2% acetone solution, and the activated CyI was added dropwise into the above solution, followed by constant stirring at room temperature overnight. The product was purified by dialysis (MWCO 1000) against distilled water for 3 d. After dialysis, the solution was self-assembled with sonication at P=300 W for 60 times and then centrifuged. The resultant nanoparticles Chitosan-CyI (CC) were kept at room temperature for further research.

### Preparation of MnO_2_-Chitosan-CyI (MCC)

MnO_2_-Chitosan-CyI (MCC) were obtained after the biomineralization growth of MnO_2_ in the presence of CC. The procedure was following the protocol reported previously 21. Briefly, CC was dissolved in water, and 0.1 mL MnCl_2_ solution (2.52 mg) was slowly added under continuous stirring. Then, solution pH was adjusted to 11 by NaOH (1.0 M). After stirring for another 2 h, the mixture was further dialyzed against distilled water to remove excess precursors in order to obtain MCC nanosystem.

### Characterization

UV-Vis-NIR spectra were acquired by Beckman Coulter DU 640 spectrophotometers. Fluorolog-3 fluorometer was utilized for fluorescence spectra detection. All optical measurements were performed at room temperature. The morphology of nanocomposite was characterized by transmittance electron microscope (TEM, JEM2010, JEOL, Japan) with accelerated voltage of 200 KV by negatively staining TLs with 2% phosphotungstic acid. The hydrodynamic diameters were measured by Mastersizer Nano-ZS90 laser particle size analyzer (Malvern, British) at 25 °C. X-ray photoelectron spectroscopy (XPS) spectra were performed on an ESCALAB 250Xi instrument (Thermo Fisher Scientific, UK).

### Singlet oxygen measurements

^1^O_2_ production was evaluated based on the protocol reported previously 40. In brief, 50 μl of the 25 μM stock solution of SOSG was dissolved in methanol, and then 50 μL of PBS (pH=7.4) or 50 μL sample solutions (125 μg/ml) were added to the parallel wells of the 96 well opaque plate. Next, all wells were irradiated with light (808 nm, 0.3, 0.96 or 1.6 W/cm^2^, 5 min). The PBS solution was used as control. The samples were analyzed with Fluorolog-3 fluorometer using an excitation of 504 nm and an emission of 525 nm.

### PTT property of MCC nanosystems

Photothermal effect of MCC in the tumor microenvironment were evaluated by recording the temperature curve of the samples irradiated with an 808 nm NIR laser at different power (0.3, 0.96 or 1.6 W/cm^2^) as the irradiation time increases. Temperatures were quantified using a thermocouple thermometer (TES Electrical Electronic Corp, WRNK-104) at designated time intervals.

### Cell uptake study

The uptake of the nanosystems was performed in 4T1 cells. In brief, 4T1 cells (3 × 10^5^ cells per mL) were seeded in a confocal Petri dish and incubated at 37 °C for 24 h. Then the medium was replaced by sample solution with culture media and incubated for different time periods (1 h, 2 h, 4 h and 8 h). After incubation, the adherent cells were washed with PBS thrice and then imaged using laser confocal microscopy (Nikon A1R MP, Japan).

### Intracellular ROS/Hypoxia assay

Oxidative stress and hypoxia assay kit were used to detect intracellular ROS and hypoxia production. Hypoxic condition was achieved by incubation in hypoxia chamber containing 1% O_2_, 5% CO_2_, and 95% N_2_ gas at 37 °C for 4 h. 4T1 cells were seeded in cell culture glass dishes at a density of 10^6^ cells in an oxygen-deficient environment. The medium was replaced with different sample solution at the CyI concentration of 25 μM with or without H_2_O_2_ (50 µM, or GSH (2 mM)) for 4 h. After washing with cold PBS, cells were irradiated with a NIR laser (808 nm, 0.3 or 0.96 W/cm^2^) for 3 min and incubated with fresh media containing Hypoxia/oxidative stress detection probes for 4 h and then observed using laser confocal microscopy. The hypoxic fluorescence signal (red) and ROS fluorescence signal (green) was detected with an excitation wavelength of 561 nm and 488 nm, respectively. In parallel, the cells without NIR dyes were as control.

### *In vitro* PTT evaluation

Intracellular heat generation of MCC was evaluated by first treating 4T1 cells with different sample solutions (125 µg/mL) for 4 h. After washing by cold PBS, cells were collected and exposed to a NIR laser for 3 min (808 nm, 0.3 W/cm^2^ or 0.96 W/cm^2^). During laser irradiation, the temperatures of these cells were monitored every 30 seconds using a thermocouple thermometer and thermal images were recorded using an infrared thermal imaging camera (Xtherm T3Pro, IRay, China).

### *In vitro* phototherapy study

*In vitro* cytotoxicity of MCC was first evaluated in mouse breast cancer cells 4T1 and human liver normal cell lines L-02 according to standard protocols reported previously 42. Then, to confirm the dual-modulated tumor hypoxia for enhanced phototherapy, MTT assay was carried out in 4T1 cell lines. Briefly, cells seeded in 96-well microliter plates (2.5×10^3^ cells per milliliter) were incubated with MCC in the presence of H_2_O_2_ (50 µM) or GSH (2 mM) for 4 h. After washing with cold PBS, cells were irradiated with an 808 nm NIR laser at designed power density for 3 min and incubated for another 24 h. The cell viability rate was calculated as described above.

The enhanced phototherapy effects of nanosystems on 4T1 cells were further verified using Calcein AM and propidium iodide (PI) co-staining. 4T1 cells were incubated with different sample solutions and irradiated as stated above. Then, the cells were stained with a mixed solution of calcein AM and PI at room temperature for 15 min and examined under the laser scanning confocal microscopy. The excitation wavelength was set at 490 nm for calcein AM and 535 nm for PI.

### *In vitro* immunomodulation of tumor-associated macrophage (TAMs) phenotype

For macrophage polarization, the RAW264.7 cells were seeded in a six-well plate (5×10^5^ cells/well in 1 mL DMEM) and cultured overnight. The models of classically (M1-type) and alternatively (M2-type) activated macrophages were established by stimulating M0-type RAW 264.7 cells with LPS (1 μg/mL)/IFN-γ (50 ng/mL) and IL-4 (50 ng/mL) for 24 h, respectively. The macrophage cells with different phenotypes were collected and seeded in the four-well confocal dish and incubated overnight. After fixing with methanol for 5 min and blocking by 1% BSA in PBS for 30 min at room temperature, the cells were stained with FITC-anti-CD86 (1 μg/mL) and PE-anti-CD206 (1 μg/mL) for 1 h. The free antibodies were removed by washing several times, after which the cells were visualized using laser scanning confocal microscopy.

To investigate immunomodulation of M2-type TAMs, the macrophage cells were incubated with CyI or MCC for 24 h. The macrophage cells with different phenotypes were acquired by scraping and the cells were diluted to 5×10^5^ cells in cold PBS (200 μL) with 10 % FBS. Then FITC-anti-CD86 (1 μg) or PE-anti-CD206 (1 μg) was added into the cells suspension and incubated at room temperature for 45 min. Finally, the cells were washed three times with PBS by centrifugation (2000 rpm, 5 min) and re-suspend in 1 mL PBS for flow cytometry.

### *In vitro* inhibition of tumor cells by MCC during post-PDT period

Firstly, the RAW264.7 macrophage cells at different polarization states were prepared as previously treated by LPS/IFN-γ, IL-4, CyI and MCC, respectively. At the same time, the macrophage cells were seeded into a permeable transwell insert (1×10^5^ cells/well in DMEM). 4T1 cells were seeded onto 12-well plates at a density of 1×10^5^ cells per well. The next day, 4T1 cells were incubated with CyI or MCC for 4 h. After washing by cold PBS, 4T1 cells were irradiated with an 808 nm NIR laser at 0.96 W/cm^2^ power density for 5 min. After 24 h incubation in a carbon dioxide incubator, the medium was changed and the residual tumor cells were co-cultured with the macrophages treated by CyI and MCC in the upper chamber of transwell insert upon different polarization states (M1 or M2 type). 4T1 cells without MCC or CyI treatment were co-incubated with M1-type and M2-type macrophages stimulated by LPS/IFN-γ and IL-4, respectively. Then, according to standard manufacturer's protocols, the viability of all 4T1 cells were determined every day using a sensitive *in vitro* toxicology test kit (MTT). Finally, according to the procedure described by the manufacturer, the medium in the plate was collected at the 5th day for various cytokines detection using the ELISA kit of IL-6, IL-10 and TNF-α.

### *In vivo* biodistribution and tumor-targeting evaluation

BALB/c mice (half male and half female) (evenly, male and female animals aged 6-8 weeks, weighed 16-21 g, bred in a GLP laboratory) were used in this investigation. For* in vivo* study, 4T1 cells suspension (100 μL, 1×10 ^6^ cells) was injected into the mice subcutaneously after the mice were anesthetized by using isoflurane. When the volume of the tumor reaches100~300 mm^3^, the mice were used for *in vivo* biodistribution and targeting experiments. The tumor bearing mice were divided into 2 groups (5 mice per group), which were intravenously injected with either CyI (0.2 mL, control) or MCC (0.2 mL) via the tail vein. At the predetermined time after administration, 4T1 tumor-bearing mice were imaged using IVIS spectrum imaging system. Subsequently, the mice were sacrificed at 24 h, and the main organs as well as tumors were harvested and imaged. The corresponding fluorescence imaging was analyzed by the instrument software.

### *In vivo* evaluation of PDT/PTT effect

4T1 tumor-bearing mice were randomly divided into 5 groups (5 mice each group). Each mouse was intravenously (i.v.) injected with different sample solutions (200 μL) and treated with different therapeutic schedules: (A) saline without laser irradiation (control group); (B) laser irradiation only (0.96 W/cm^2^, 3 min, control group); (C) CyI with laser irradiation (0.96 W/cm^2^, 3 min, 1.5 mg/kg, PTT-modulate tumor hypoxia for enhanced PDT group); (D) MCC with laser irradiation (0.3 W/cm^2^, 3 min, 1.5 mg/kg equivalent to CyI, MnO_2_-modulated tumor hypoxia for enhanced PDT group); (E) MCC with laser irradiation (0.96 W/cm^2^, 3 min, 1.5 mg/kg equivalent to CyI, dual-modulated tumor hypoxia for enhanced PDT group).

To validate the intratumoral ^1^O_2_ production of MCC, SOSG was used as a fluorescence probe. Briefly, 200 μL of sample solution was mixed separately with 50 μL SOSG (25 μM) and then i.v. injected into mice. After treatment with NIR laser, all mice were sacrificed and the tumors were collected for cryo-sectioning. Finally, the tumor sections were visualized by a laser scanning confocal microscopy and the signals were semi-quantified by the Image J software.

For evaluation of PTT-activated PDT efficacy in tumor site, a thermocouple thermometer was used to record the tumors' temperature every 30 sec and an infrared thermal imaging camera was used to monitor the temperature changes of the tumors during laser irradiation.

### *In vivo* phototherapy study

4T1 tumor-bearing mice were randomly divided into 5 groups (8 each group) as described above. The tumor size and body weight of each mouse were recorded every other day. Tumor volume was calculated as length × (width)^2^ ×1/2. All mice were sacrificed and the tumors were collected 21 days after treatment. To confirm the enhanced synergistic efficacy of MCC nanosystems for tumor therapy, histological analysis of tumor tissues was performed after treatment. Tumor tissues were isolated, fixed with 10% neutral buffered formalin and embedded in paraffin (n=5). The sliced organs were stained with hematoxylin and eosin and examined by a microscope. Tumor apoptosis was also assessed using TUNEL assay and Ki67 staining according to the product instruction.

### Hypoxia Immunofluorescence staining

To study the hypoxia status in TME, Hypoyprobe-1 plus kit was used for immunofluorescence imaging. The staining protocol was followed by the product instruction. In brief, mice with 4T1 tumors were intravenously injected with saline, CyI or MCC (1.5 mg/kg equivalent to CyI), and the tumors were irradiated using an 808 nm laser (0.3 or 0.96 W/cm^ 2^, 3 min) at 24 h post-injection. Then, the tumors were surgically excised 90 min after intraperitoneal injection with pimonidazole hydrochloride (60 mg kg^ -1^), which was reductively activated in hypoxic cells and formed stable adducts with thiol (sulphydryl) groups in proteins, amino acids and peptides. The tumor anoxic zones were labeled with primary antibodies using fluorescein-conjugated mouse IgG monoclonal antibodies. Afterwards, the sections were stained with a peroxidase-conjugated anti-FITC secondary protocol and imaged by a confocal microscopy.

### PDT-induced immune response

To evaluate PDT-induced acute inflammatory response and immune response, mice were sacrificed, and tumors were collected for the immunological evaluations 5 d post-irradiation. Briefly, tumor tissues were cut into small pieces and put into a glass homogenizer containing PBS solution (pH=7.4). Next, the single-cell suspension was prepared by gentle pressure with the homogenizer without the addition of digestive enzyme. Finally, the tumor cell supernatant was collected to determine the levels of the following immune factors: tumor necrosis factor-α (TNF-α), interferon-γ (IFN-γ), interleukin-12 (IL-12), and interleukin-10 (IL-10), using ELISA assay (eBioscience, Thermo Fisher Scientific). The cytotoxic T lymphocytes (CTLs) and tumor-associated macrophages (TAMs) were quantified by flow cytometry. CD3^+^ and CD8^+^ cells were defined as CTLs. CD11b^+^F4/80^+^ and CD11b^+^F4/80^+^CD206^+^ cells were defined as macrophages and M2 phenotype macrophages, respectively. Therefore, CTLs were stained with anti-CD3-APC and anti-CD8-PE antibodies, and TAMs were stained with anti-CD206-FITC, anti-CD11b-PE, and anti-F4/80-AlexaFluor 647 antibodies.

### Statistical Analysis

Data was expressed as mean±standard deviation. Statistical analysis was performed by students' t-test with statistical significance assigned for P value of <0.05.

## Supplementary Material

Supplementary figures and equation.

### Supporting Information

XPS, size distribution and zeta potential of MCC; Average size changes of MCC within 30 days; Temperature change curves of MCC in solutions upon 808 nm NIR irradiation; Cell viability of MCC in L-02 and 4T1 cells; Cell viability of 4T1 cells after incubation with different concentration MCC under different therapy; Pharmacokinetics of MCC in plasma and tissues. Mean fluorescence of ROS generation in SOSG-stained tumor sections in 4T1 tumor-bearing mice after various treatments; Temperature changes inside the tumor treated with different samples and exposed to 808 nm NIR laser light; Tumor photos after different treatments in Day 21. Images of mice before and after CC or MCC treatment upon NIR irradiation in bilateral tumor model.

## Abbreviations

PDT: photodynamic therapy
PTT: photothermal therapy
NIR: near-infrared
PSs: photosensitizers
ROS: reactive oxygen species
TME: tumor microenvironment
GSH: glutathione
MCC: MnO_2_@Chitosan-CyI
CC: Chitosan-CyI
CTL: cytotoxic T lymphocyte
