# Supplementary material for: Tumor Microenvironment-triggered Nanosystems as dual-relief Tumor Hypoxia Immunomodulators for enhanced Phototherapy
Source: Theranostics. 2020 Jul 13;10(20):9132–52. doi: 10.7150/thno.46076 (PMC7415819; doi:10.7150/thno.46076)
Supplement: Supplementary file 1 — Supplementary figures and equation. [file thnov10p9132s1.pdf]

Tumor microenvironment-triggered nanosystems as dual-relief tumor hypoxia immunomodulators for enhanced phototherapy

Zijun Shen [1], Junfei Xia [2], Qingming Ma [1], Wei Zhu [3], Zhen Gao [1], Shangcong Han [1], Yan Liang [1], Jie Cao [1,*], Yong Sun [1]

[1] Department of Pharmaceutics, School of Pharmacy, Qingdao University, Qingdao, China, 266021

[2] Department of Electrical and Computer Engineering, Tufts University, Medford, MA, 02155

[3] Department of Pharmacology, School of Pharmacy, Qingdao University, Qingdao, 266021, China

* Address correspondence to caojie0829@qdu.edu.cn

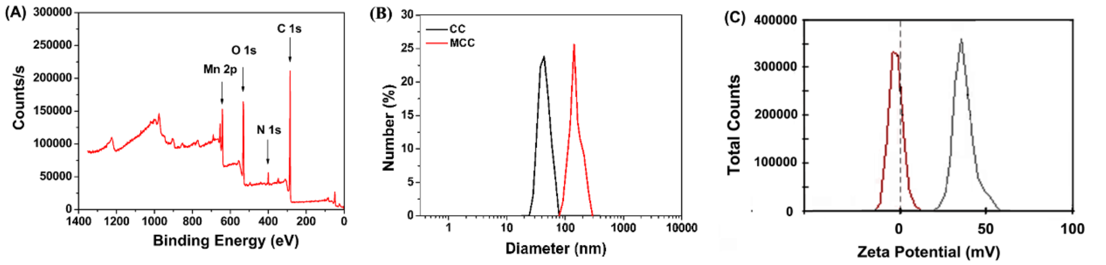

Figure S1 (A) XPS of MCC nanosystem; Size distribution (B) and zeta potential (C) of CC and MCC

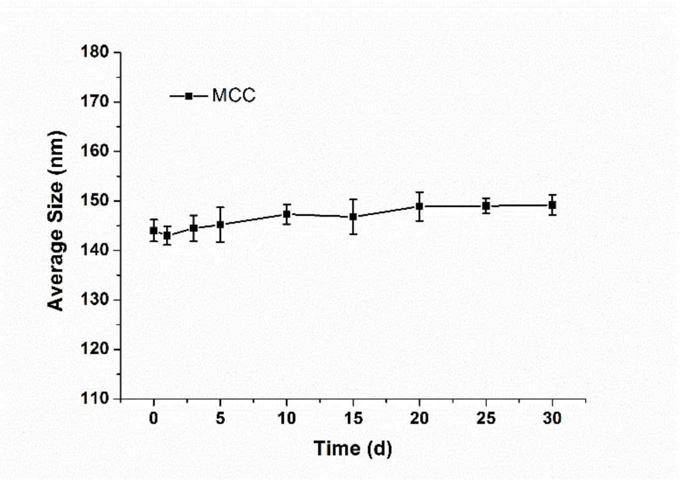

Figure S2 Average size changes of MCC within 30 days.

Equation S1:

$$\eta = \frac{\hbar A \Delta T_{max} - Q_s}{I(1 - 10^{-A_\lambda})}$$

where h is the heat transfer coefficient, A is the surface area of the container, ΔTmax is the temperature change of the CyI or Cy7 solution at the maximum steady-state temperature, I is the laser power, Aλ is the absorbance of CyI or Cy7 at 808 nm, Qs is the heat associated with the light absorbance of the solvent, which is measured independently using pure water without CyI or Cy7.

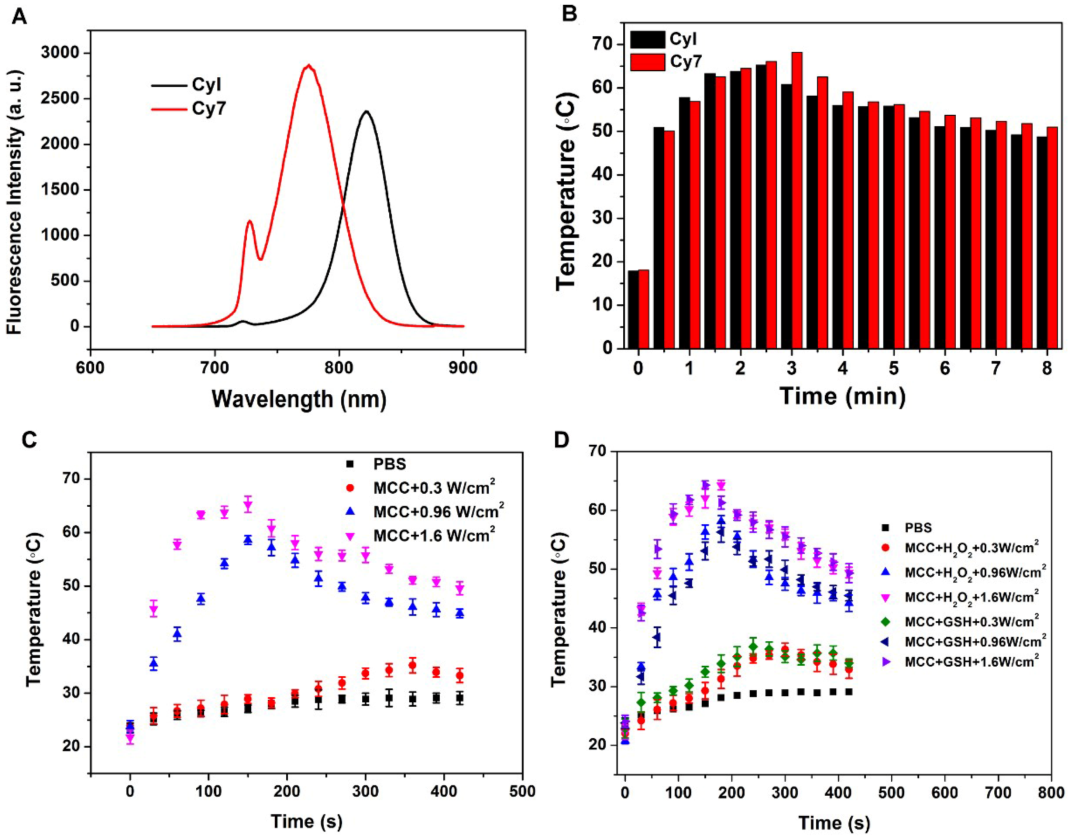

Figure S3 (A) Fluorescence spectra of CyI and Cy7; (B) Temperature change curves of CyI and Cy7 aqueous solution exposed to the 808 nm NIR laser at a power density of 1.6 W/cm^2; (C) Temperature change curves of MCC exposed to the laser (808 nm, 0.3, 0.96, 1.6 W/cm^2) under different irradiation time; (D) Temperature change curves of MCC with 10 mM GSH or 50 μM H_2O_2 solution by varying laser power density (808 nm, 0.3, 0.96 or 1.6 W/cm^2) under different irradiation time.

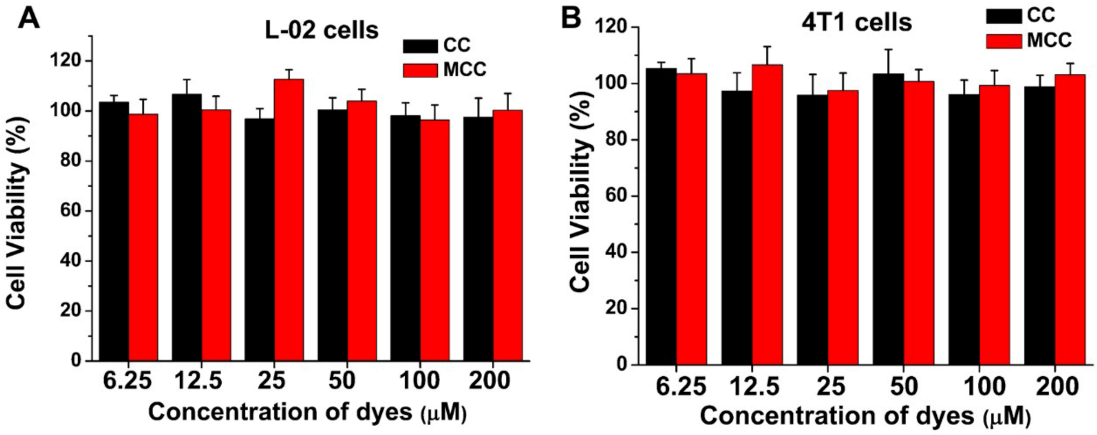

Figure S4 Cell viability of MCC in both mouse breast cancer cells 4T1 and human liver normal cell lines L-02 (with CyI concentration of 6.25, 12.5, 25, 50, 100 and 200 μM).

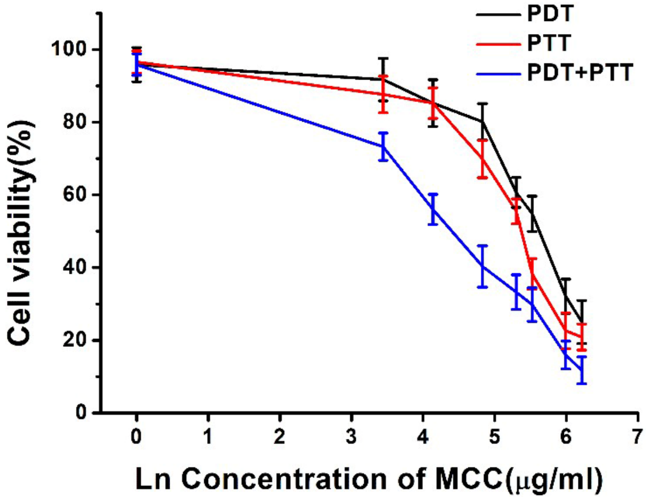

Figure S5 The cell viability of 4T1 cells after incubation with different concentration MCC under different therapy.

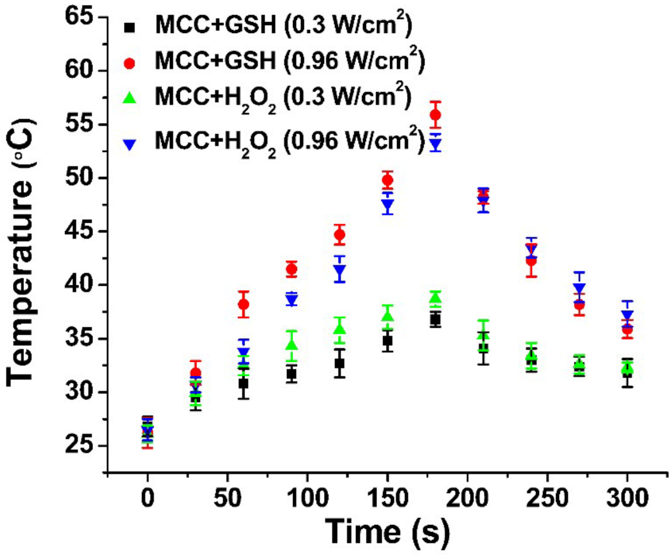

Figure S6 Temperature changes of 4T1 cells treated with MCC in the presence of GSH or H_2O_2 upon different NIR irradiation power (808 nm, 0.3 or 0.96 W/cm^2). Untreated cells and non-treated cells exposed to NIR light were as control. The data was represented as mean ± SD.

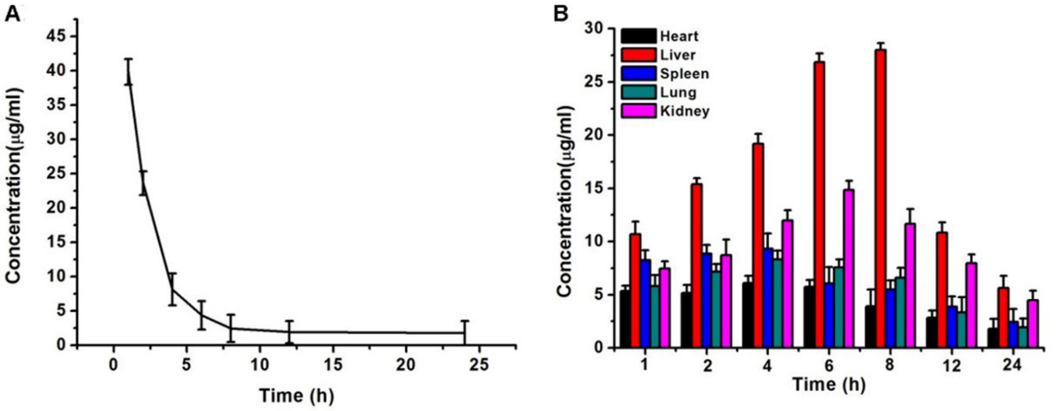

Figure S7 (A) Plasma concentration curve after intravenous MCC; (B) In vivo tissue distribution of MCC.

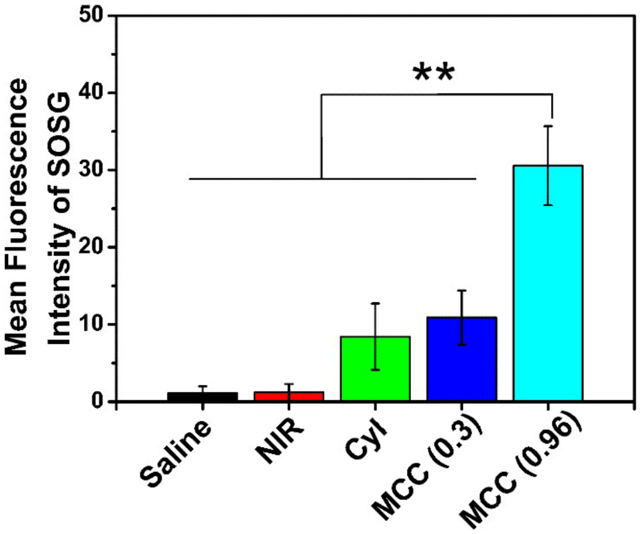

Figure S8 Mean fluorescence of ROS generation in SOSG-stained tumor sections in 4T1 tumor-bearing mice after various treatments.

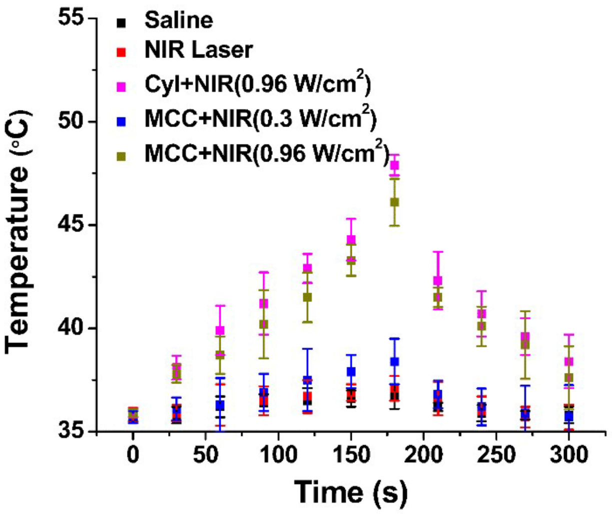

Figure S9 Temperature changes inside the tumor treated with different samples and exposed to

NIR laser light (808 nm, 0.3 or 0.96 W/cm^2, 3 min). Untreated tumor and tumor exposed to NIR light were as control.

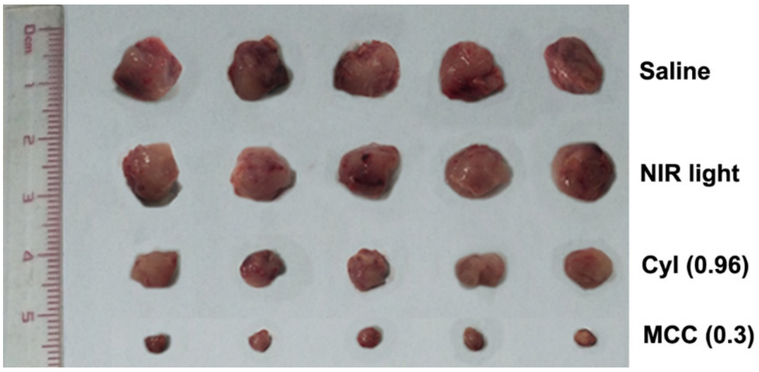

Figure S10 Tumor pictures after different treatments in Day 21

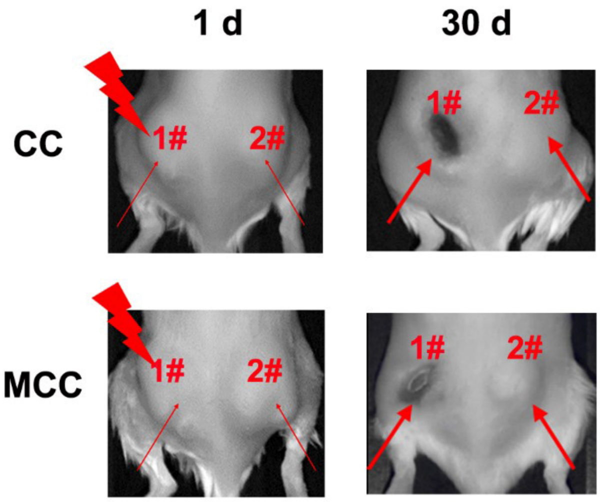

Figure S11 Images of mice before and after CC or MCC treatment upon NIR irradiation
